# Detection of Germline Mutations in a Cohort of 139 Patients with Bilateral Breast Cancer by Multi-Gene Panel Testing: Impact of Pathogenic Variants in Other Genes beyond *BRCA1/2*

**DOI:** 10.3390/cancers12092415

**Published:** 2020-08-25

**Authors:** Daniele Fanale, Lorena Incorvaia, Clarissa Filorizzo, Marco Bono, Alessia Fiorino, Valentina Calò, Chiara Brando, Lidia Rita Corsini, Nadia Barraco, Giuseppe Badalamenti, Antonio Russo, Viviana Bazan

**Affiliations:** 1 Department of Surgical, Oncological and Oral Sciences, Section of Medical Oncology, University of Palermo, 90127 Palermo, Italy; fandan@libero.it (D.F.); clarissafilorizzo@gmail.com (C.F.); marcobono29@gmail.com (M.B.); alessiafiorino94@gmail.com (A.F.); valentinacalo74@gmail.com (V.C.); chiabra92@libero.it (C.B.); lidia.corsini@gmail.com (L.R.C.); barraconadia@gmail.com (N.B.); giuseppe.badalamenti@unipa.it (G.B.); 2 Department of Biomedicine, Neuroscience and Advanced Diagnostics (Bi.N.D.), Section of Medical Oncology, University of Palermo, 90127 Palermo, Italy; lorena.incorvaia@unipa.it (L.I.); viviana.bazan@usa.net (V.B.)

**Keywords:** *ATM*, *BRCA1*, *BRCA2*, breast cancer, bilateral breast cancer, *CHECK2*, germline pathogenic variants, multi-gene panel testing, *PALB2*, *PTEN*

## Abstract

**Simple Summary:**

Many bilateral breast cancer patients with increased hereditary susceptibility to breast cancer result negative for *BRCA1* or *BRCA2* pathogenic variants and, thus, need a further genetic testing through a broader gene panel. Some patients with negative test result for *BRCA1/2* pathogenic variants may harbor pathogenic variants in other breast cancer susceptibility genes, including *ATM*, *CHEK2*, *PALB2*, *PTEN*, *TP53*. Of course, the use of a multi-gene panel provides clinicians more information through a single test. Therefore, we focused on potential clinical impact of a NGS-based multi-gene panel testing in bilateral breast cancer patients, in order to evaluate the utility of perform a most comprehensive genetic analysis in these subjects, regardless the criteria concerning personal and family history of cancer established by the current guidelines. Our study revealed that the use of a NGS-based multiple-gene panel testing could increase the detection rates of germline alterations in bilateral breast cancer patients.

**Abstract:**

Patients with unilateral breast cancer (UBC) have an increased risk of developing bilateral breast cancer (BBC). The annual risk of contralateral BC is about 0.5%, but increases by up to 3% in *BRCA1* or *BRCA2 pathogenic variant* (PV) carriers. Our study was aimed to evaluate whether all BBC patients should be offered multi-gene panel testing, regardless their cancer family history and age at diagnosis. We retrospectively collected all clinical information of 139 BBC patients genetically tested for germline PVs in different cancer susceptibility genes by NGS-based multi-gene panel testing. Our investigation revealed that 52 (37.4%) out of 139 BBC patients harbored germline PVs in high- and intermediate-penetrance breast cancer (BC) susceptibility genes including *BRCA1*, *BRCA2*, *PTEN*, *PALB2*, *CHEK2*, *ATM*, *RAD51C*. Nineteen out of 53 positively tested patients harbored a PV in a known BC susceptibility gene (no-*BRCA*). Interestingly, in the absence of an analysis performed via multi-gene panel, a significant proportion (14.4%) of PVs would have been lost. Therefore, offering a NGS-based multi-gene panel testing to all BBC patients may significantly increase the detection rates of germline PVs in other cancer susceptibility genes beyond *BRCA1/2*, avoiding underestimation of the number of individuals affected by a hereditary tumor syndrome.

## 1. Introduction

Breast cancer (BC) is the most commonly diagnosed female malignancies worldwide [1]. Women with unilateral breast cancer (UBC) have an increased risk of developing bilateral breast cancer (BBC) [2]. The cumulative incidence rate of developing contralateral BC at 10 years is about 3.4% for UBC patients [1,3]. In recent years, the increasing BC incidence rates observed through screening programs, improved treatment, and growing life expectancy have resulted in the early detection of increasing incidence of developing BBC [1,4]. Multiple criteria are associated with increased risk of second primary BC, including an early age of onset, hormone receptor-positivity of the initial tumor, race and ethnicity, as well as presence of known pathogenic variants (PVs) in hereditary cancer-associated genes [5]. Although BC is frequently a sporadic tumor (75–80%), approximately 15–20% are familial type and about 5–10% of cases are hereditary, caused by germline PVs in specific breast cancer-associated genes [5,6,7,8]. The two genes mainly involved in the BC genetic predisposition are *BRCA1*, located on chromosome 17 [9] and *BRCA2*, located on chromosome 13 [10]. Generally, the cumulative risk of developing BC by age 80 years has been observed to be 72% for germline *BRCA1* PV carriers and 69% for germline *BRCA2* PV carriers, respectively [11]. Specifically, the annual risk of contralateral BC is about 0.5%, but increases by up to 3% in *BRCA1* or *BRCA2* PV carriers, increasing 10-year cumulative risk up to 13–40% [1,12,13]. In a recent study, Kuchenbaecker et al. [11] have reported that the 20-year cumulative risk for contralateral BC, after a first BC diagnosis, was 40% for germline *BRCA1* PV carriers and 26% for germline *BRCA2* PV carriers. Moreover, germline *BRCA1* PV carriers showed a 15–45% risk of developing ovarian cancer (OC) or tubal carcinoma, whereas germline *BRCA2* PV carriers exhibited a 10–20% risk of developing OC [14,15,16].

Currently, both the National Comprehensive Cancer Network (NCCN) guidelines and European Society for Medical Oncology (ESMO) guidelines recommend genetic testing for *BRCA1* and *BRCA2* for women with multiple primary breast cancers, if first diagnosis was ≤50 years old [17,18]. Additionally, the Italian guidelines, established by the Italian Medical Oncology Association (AIOM), provide the same recommendation, also suggesting to perform the genetic tests on patients with a personal history of BC diagnosed before age 50 years with a family history of one or more first-degree relatives with BBC. With the increase in the acquisition of multi-gene panels, genetic testing used for detecting alterations in other genes beyond *BRCA1* and *BRCA2* has become more prevalent [5,7]. Moreover, the role of genetic testing in BC is rapidly changing, becoming an interesting area of research. In fact, although *BRCA1* and *BRCA2* are the two high-penetrance genes mainly correlated with increased risk of hereditary BC, several studies have identified many other susceptibility genes for BC [5]. Recently, Tebaldi et al. [19] found high rates of PVs in another BC susceptibility gene, named *PALB2*, in individuals with BBC, also observing an overall higher rate of BBC in patients with non-*BRCA* PVs than those that were *BRCA1*- or *BRCA2*-positive. Additionally, a recent study has defined high-risk BC genes as those with a BC odds ratio >5.0 (*BRCA1*, *BRCA2*, *PALB2*, *STK11*, *TP53*, *PTEN*, and *CDH1*) [20]. In particular, *PALB2* and *PTEN* are considered high-risk genes with lifetime risk above 40%. Low/moderate-risk BC genes have been reported as those with a BC odds ratio between 2.0 and 5.0 (*ATM*, *CHEK2*, and *NBN*) [20]. The identification of germline PVs in high-risk individuals allowed an increase in surveillance and earlier implementation of the risk-reduction strategies [5,21,22]. However, germline PVs detected in low/moderate-risk BC genes are also interesting and may have a significant clinical impact [23]. It was observed that approximately 1% of women of European origin affected by BC harbor germline *CHEK2* PVs, including the most commonly detected founder mutation named 1100delC [24]. The probability of carrying a *CHEK2* PV is high in the Netherlands [24], where the risk of a contralateral BC was estimated to be increased by up to 6.5-fold for women with a *CHEK2* 1100delC mutation [25].

Based on a Breast Cancer BRCA System database retrospectively collected at University Hospital Policlinico “P. Giaccone” of Palermo, the aim of this work was to describe the typology and gene location of germline PVs detected both in *BRCA1/2* and other susceptibility genes, in order to investigate the prevalence of different inherited genetic variants in BBC patients and evaluate the utility of performing a NGS-based multi-gene panel testing in these individuals. This information could be useful and interesting in order to decide if the use of a multi-gene panel testing is recommendable for BBC patients, regardless their cancer family history and age at diagnosis.

## 2. Results 

### 2.1. Clinical Features of Bilateral Breast Cancer Patients

In total, 139 BBC patients (137 of which females and two males) were recruited and studied over a period ranging from October 2015 to June 2020 at the “Regional Center for the prevention, diagnosis and treatment of rare and heredo-familial tumors of adults” of the Section of Medical Oncology of the University Hospital Policlinico “P. Giaccone” of Palermo. 

Ninety-three patients had metachronous BC, and 46 had synchronous BC. The average age of the first malignancy diagnosis was 45 years (range: 21–77 years). The average age of the second tumor was 51 years (range: 21–80 years). The average interval between the first and second BC diagnosis was 4 ± 6.08 years. In our study, 88 out of 139 patients had a second contralateral tumor within 5 years of the first tumor diagnosis, 27 subjects between 6 and 10 years, while only 24 individuals developed second tumor 10 years after the first diagnosis. 

Considering the general clinical features and family history of the study population, 53 women (38.1%) were postmenopausal, five women (3.6%) had previous ovarian cancer, 82 patients (59%) had a BC family history, 44 (31.6%) of which with one relative and 38 (27.4%) with two or more family members affected by BC. Moreover, 11 patients (7.9%) had a family history of ovarian cancer, eight (5.7%) of which with one relative and three (2.2%) with two or more family members affected by ovarian cancer (Table 1).

As regards the tumor histology of first BC, 96 (69%) out of 139 patients had ductal carcinoma in situ (DCIS), 13 (9.4%) invasive ductal carcinoma (IDC), 12 (8.6%) invasive lobular carcinoma (ILC), and finally 18 patients showed other types of BC. Considering the histology of the second tumor, 16 patients (11.5%) had DCIS, 92 (66.2%) IDC, 15 (10.8%) ILC, 16 patients (11.5%) other types of BC. Finally, both for the first and second diagnosis, the most representative molecular phenotypes of BC were luminal B/HER2- (35.3% and 25.2%, respectively) and luminal A (20.1% and 25.1%, respectively) (Table 1).

### 2.2. Detection of Germline Pathogenic Variants in Cancer Susceptibility Genes by Multi-Gene Panel Testing

All 139 probands were genetically tested for germline PVs in different cancer susceptibility genes, including *BRCA1* and *BRCA2*, by NGS-based multi-gene panel testing. The mutational screening showed that 52 (37.4%) out of 139 genetically tested BBC patients harbored germline PVs (class V) in several high- and intermediate-penetrance BC susceptibility genes including *BRCA1* and *BRCA2*, one patient showed a heterozygous PV in *MUTYH* gene, 27 (19.4%) patients were carriers of variants of uncertain significance (VUS; class III), six of which in *BRCA1/2*, whereas 59 (42.5%) showed no genetic variants (classes III, IV, and V) (Table 1). 

Our analysis revealed that 33 (62.3%) out of 53 PV-positive BBC patients have been shown to harbor germline *BRCA1/2* PVs, 13 (24.5%) of which in *BRCA1* and 19 (35.8%) in *BRCA2*, and only one (1.9%) in both genes (double heterozygosity for *BRCA1* and *BRCA2* PVs) (Table 2). Nineteen (35.8%) out of 53 individuals positively tested by multi-gene panel have been shown to carry a PV in a known BC susceptibility gene (no-*BRCA*). In particular, five (9.4%) out of 53 PV-positive BBC patients have been shown to harbor *CHEK2* PVs, eight patients (15.1%) showed *PALB2* PVs, three individuals (5.7%) carried *ATM* PVs, only one patient (1.9%) exhibited a *PTEN* PV, while PVs in *RAD51C* were detected in two (3.8%) triple-negative BC patients (Table 3). No PVs were detected in MMR genes (*MLH1*, *MSH2*, *MSH6*, *PMS2*, and *EPCAM*).

**Figure 1:**
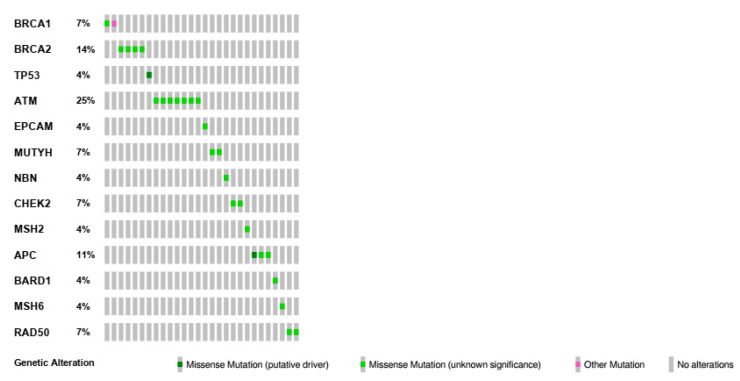
Distribution and frequency of variants of uncertain significance (VUS) identified in different genes of bilateral breast cancer patients. The OncoPrint, showing the identified VUS by heatmap, was obtained by the informatic tool Mutation Mapper-cBioPortal for Cancer Genomics.

Additionally, based on the classification criteria developed by the Evidence-based Network for the Interpretation of Germline Mutant Alleles (ENIGMA) consortium and according to IARC recommendations [26], 28 different genetic variants detected in 27 BBC patients were classified as VUS (class III). The VUS analysis showed that these variants were mainly located on 13 genes, including *ATM*, *BRCA1*, *BRCA2*, *RAD50*, *CHEK2*, *APC*, *BARD1*, *TP53*, etc. (Table 4). Most VUS were found on the *ATM* gene in a heterozygous state (seven different variants, 25%), but to a lesser degree also on *BRCA2* (14%), *APC* (11%), and *BRCA1* (7%) (Figure 1). Finally, our analysis also showed that some patients were carriers of more than one VUS on different genes. 

### 2.3. Type and Gene Location of Genetic Variants in Bilateral Breast Cancers

The most frequently detected PVs in most *BRCA*-positive carriers were c.4964_4982del (p.Ser1655fs) and c.3226_3227AG [1] (p.Gly1077fs) for *BRCA1*, and c.1238del (p.Leu413fs) for *BRCA2*. The most common Sicilian founder variant named c.4964_4982del [27] resulted as among the most recurrent *BRCA1* PVs in BBC patients, since this variant was detected in four families, whereas the other most frequent *BRCA1* PV named c.3226_3227AG [1] was reported as a founder mutation both in Italy (Tuscany) and Norway [28].

Additionally, the *BRCA2* PV named c.1238del, frequently detected in the luminal B molecular subtypes observed in BBCs, could be a good candidate for becoming a potential variant with founder effect in Sicily, but further in-depth studies are needed.

Interestingly, other two *BRCA2* PVs named c.9098_9099insA (p.Gln3034fs) and c.8594T > A (p.Leu2865Ter), albeit found in only one BBC proband, respectively, were detected in 18 and 10 family members, respectively.

As regards the analysis of other moderate-risk susceptibility genes, the c.1100delC variant, as already previously demonstrated by other authors [29,30], has been shown to be the most recurrent CHEK2 PV in our study population, since it was detected in five BBC patients. Interestingly, we found that this variant mainly correlates with a luminal A/B phenotype, estrogen receptor positivity >60%, and progesterone receptor positivity between 20% and 60%.

Although *PALB2* has been shown to be the most frequently altered gene in BBC patients, besides *BRCA1* and *BRCA2*, however four different variants were identified. Among no-*BRCA* genes, also heterozygous *ATM* resulted as altered in BBC individuals, showing three different genetic variants, including a large genomic rearrangement (LRG). The *ATM* PVs harbored by all three BBC patients were mainly observed in the luminal B tumor phenotype. Finally, a monoallelic PV in *MUTYH* gene, whose homozygous alterations are usually correlated with *MUTYH*-associated colon polyposis (MAP) syndrome and colorectal cancer [31], was unusually observed in one BBC patient.

Our study also aimed to evaluate the typology and gene location of germline PVs in BBCs, in order to investigate potential associations between specific PVs and bilaterality in BC.

**Figure 2:**
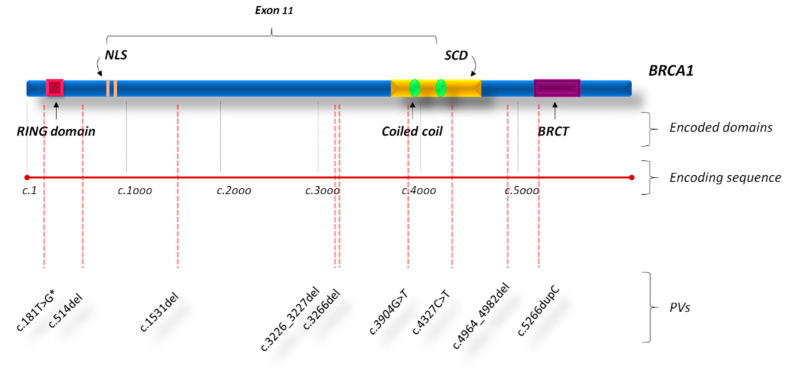
BRCA1 functional domains and gene location of *BRCA1* PVs in bilateral breast cancer patients. Abbreviations: BRCT, BRCA1 C-terminus domain; NLS, nuclear localization sequence; PVs, pathogenic variants; SCD, serine cluster domain. * This PV is present together with the *BRCA2* PV named c.8331+2T>C in one patient showing double heterozygosity for *BRCA1* and *BRCA2* PVs.

As regards the gene location of *BRCA1* PVs detected in BBC patients of our study cohort, most of observed *BRCA1* PVs (6/9) were frameshift mutations and were equally distributed along the entire gene sequence, in particular inside three hypothetical cluster regions present in the BRCA1 protein structure which includes the RING domain at the N-terminus, a region encoded by exon 11, and BRCT domain near the C-terminus. Five out of nine *BRCA1* PVs were detected in the exon 11 of BBC patients (nucleotides: 1650–4446; codons: 512–1143), whereas two in the sequence corresponding to the BRCT repeats (nucleotides: 5083–5382; codons: 1655–1756) and the other two in the RING domain (nucleotides: 300–633; codons: 61–172) (Figure 2).

**Figure 3:**
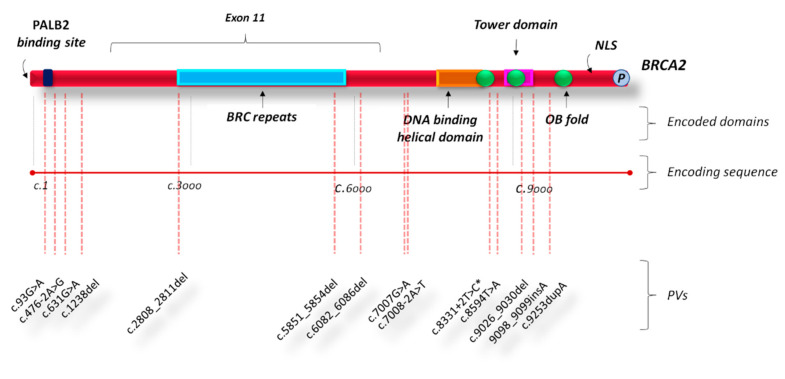
BRCA2 functional domains and gene location of *BRCA2* PVs in bilateral breast cancer patients. Abbreviations: NLS, nuclear localization sequence; OB, oligonucleotide binding; PVs, pathogenic variants. * This PV is present together with the *BRCA1* PV named c.181T>G in one patient showing double heterozygosity for *BRCA1* and *BRCA2* PVs.

Conversely, most of observed *BRCA2* PVs were mainly localized inside three other putative cluster regions present in the BRCA2 protein structure which includes the PALB2 binding site at the N-terminus, BRC repeats (located within the exon 11), and DNA binding helical domain near the C-terminus. Four *BRCA2* PVs were located at the N-terminus, near the PALB2 binding site (nucleotides: 321–1466; codons: 31–413), whereas three inside the exon 11 (nucleotides: 3036–6310; codons: 938–2028), two of which within the BRC repeats, and, finally, five variants were detected at the C-terminus, near DNA binding helical domain (nucleotides: 8559–9481; codons: 2865–3085) (Figure 3). Half of the *BRCA2* PVs found in BBC patients were frameshift mutations, three were intronic variants (IVS), while two were missense and two nonsense.

## 3. Discussion

The incidence of BBC is slightly higher, 3%, than all BCs. More precisely, synchronous tumors (contemporary bilaterality) represent 0.6%, while those metachronous constitute 2.2% [32,33].

Some of these BBCs have been shown to be related to the Hereditary Breast and Ovarian Cancer (HBOC) syndrome, since the annual risk of contralateral BC significantly increases in *BRCA1* or *BRCA2* PV carriers [13]. However, many BBC patients with increased hereditary susceptibility to BC result negative for *BRCA1* or *BRCA2* PVs and, thus, need further genetic testing through a broader gene panel. Some patients with negative test result for *BRCA1/2* PVs may harbor a still undiscovered PV in these genes, probably due to the sequencing limitations, or PVs in other cancer susceptibility genes, including *ATM*, *CHEK2*, *PALB2*, *PTEN*, *TP53*, and others, in 3–4% of cases [34,35]. For this reason, today, thanks also to the progress obtained by next-generation sequencing (NGS) technology which revolutionized the clinical approach to genetic testing, the use of multi-gene panels containing several susceptibility genes beyond *BRCA1/2* is becoming increasingly frequent [36,37]. Recent studies showed that, because of overlapping phenotypes, a significant number of PVs, which would be missed if individually tested for HBOC, Cowden syndrome, Li–Fraumeni syndrome, Lynch syndrome, or other hereditary cancer syndromes, could be more easily detected by multi-gene panel testing. Therefore, single gene panels covering all these syndromes rather than individual syndrome-specific panels were generated [38]. Of course, the use of a multi-gene panel provides clinicians more information about one or more syndromes in a single test [39]. The clinical significance of comprehensive multi-gene panels useful in the clinical management of hereditary breast and ovarian tumors was recently investigated in numerous studies [36,40]. However, today, the debate about the choice of genes to include for each syndrome and identification of specific groups of high-risk patients who should be offered multi-gene panel testing still remains open. Indeed, to date, no well-defined guidelines have been written to identify criteria for selecting the most suitable patients to perform multi-gene panel testing on.

In this work, we retrospectively collected and analyzed all clinical information of 139 BBC patients who have been genetically tested for germline PVs in different cancer susceptibility genes, including *BRCA1* and *BRCA2*, by NGS-based multi-gene panel testing. This study aimed to evaluate whether all BBC patients should be offered multi-gene panel testing, regardless of their cancer family history and age at diagnosis.

Our investigation revealed that 52 (37.4%) out of 139 genetically tested BBC patients were shown to carry germline PVs in high- and intermediate-penetrance BC susceptibility genes such as *BRCA1*, *BRCA2*, *PTEN*, *PALB2*, *CHEK2*, *ATM*, *RAD51C*. Interestingly, a noteworthy correlation between PVs in *PALB2* or *CHEK2* and BBCs was observed. In addition, our study showed that *CHEK2* PVs correlate with a luminal A/B phenotype and *ATM* PVs with a luminal B subtype. Lower percentages of PVs were found also in *PTEN* and *RAD51C* genes. Furthermore, 19.4% of patients with BBC (27/139) were shown to be carriers of VUS in several cancer susceptibility genes.

Finally, we observed that most of BBC-related germline *BRCA1/2* PVs were frameshift mutations mainly located within the exon 11 for *BRCA1*, and near the PALB2 binding site (at the N-terminus) and the DNA binding helical domain (at the C-terminus) for *BRCA2*.

Our work showed that a deeper genetic analysis through NGS-based multi-gene panel testing may help us to identify PVs in high- and moderate-risk susceptibility genes different from most common *BRCA1* and *BRCA2* genes, allowing to stratify a considerable portion of hereditary BBC patients who may benefit from the screening programs, active surveillance strategies, or risk-reducing surgery interventions, where necessary. In addition, this information could allow to identify family members with a greater risk of developing BC (or other tumors) and implement prevention and surveillance programs for these subjects. However, larger study cohorts are needed in order to determine more precise rates of PVs in BBC patients with previously negative *BRCA* genetic testing. Finally, our study has shown been to be compatible with the recommendations of the current NCCN guidelines, providing a more cost-effective cancer risk evaluation compared with a gene-by-gene approach.

## 4. Patients and Methods

### 4.1. Study Population

A retrospective cohort study was performed at the “Sicilian Regional Center for the Prevention, Diagnosis and Treatment of Rare and Heredo-Familial Tumors” of the Section of Medical Oncology of University Hospital Policlinico “P. Giaccone” of Palermo. All patients diagnosed with BBC, enrolled from October 2015 to June 2020 at a single institution, underwent germline genetic testing by multi-gene panel including: (i) high-risk BC susceptibility genes such as *BRCA1*, *BRCA2, CDH1, PALB2, PTEN, STK11*, and *TP53*; (ii) moderate-risk BC susceptibility genes such as *ATM*, *BARD1*, *BRIP1*, *CHEK2*, *NBN*, *RAD50*, *RAD51C*, and *RAD51D*; (iii) cancer predisposition genes related to other hereditary tumor syndromes such as *MLH1*, *MSH2*, *MSH6*, *PMS2*, *EPCAM*, *APC*, *MUTYH*. The only criterion used for performing genetic testing was the presence of a BBC in patients above age 18 years, regardless of criteria concerning the cancer family history and age at diagnosis established by the guidelines.

Genetic counselling was carried out by a multidisciplinary team mainly consisting of an oncologist, a geneticist, and a psychologist. The information concerning the personal and familial history of tumor, family geographical origin, age of cancer diagnosis, disease stages (I–IV), histological tumor subtype, molecular phenotype, risk factors were anonymously recorded for all patients who previously provided a written informed consent. The estrogen-receptor (ER), progesterone-receptor (PgR), HER2-receptor status (HER2), Ki67 status, and histological grade (Grades I, II, and III) of the primary and secondary tumors were extrapolated from histological report of diagnostic core biopsies or tumor resections for clinical use.

When a pathogenic or likely pathogenic variant was identified in a patient, the genetic test result was considered informative. Conversely, genetic test result was defined not informative, when no pathogenic or likely pathogenic variant was detected, but its presence could not be excluded, or a VUS to which it was not possible to attribute a risk value was detected.

Patients carrying a germline PV in any of analyzed genes were addressed to enhanced screening programs and/or risk-reducing surgical strategies by an oncologist with expertise in cancer genetics. Targeted genetic testing was proposed and extended to the first-degree family members of patients harboring a mutation, after providing informed consent.

### 4.2. Sample Collection and Next-Generation Sequencing Analysis by Multi-Gene Panel

Peripheral blood samples were collected from BBC patients. Genomic DNA was isolated from the peripheral blood using the DNeasy^®^ Blood Kit (QIAGEN), quantified by Qubit^®^ 3.0 fluorometer (Thermofisher Scientific, Waltham, MA, USA) and its quality was assessed by using 2100 Bioanalyzer (Agilent Technologies, Santa Clara, CA, USA). We used 4 ng of DNA to prepare the barcoded library using a Multi-Gene Hereditary Cancer Panel Testing named HEVA SCREEN (4bases SA) that has allowed to perform a mutational screening on 22 genes involved in risk of hereditary breast, ovarian and colorectal cancer, and other inherited tumor syndromes (*ATM, APC, BARD1, BRCA1, BRCA2, BRIP1, CDH1, CHEK2, EPCAM, MLH1, MSH2, MSH6, MUTYH, NBN, PALB2, PMS2, PTEN, RAD50, RAD51C, RAD51D, STK11*, and *TP53)*. The kit consists of three multiplex PCR primer pools. We used 20 ng of DNA per primer pool for multiplex PCR amplification, followed by barcode ligation and purification with Agentcourt AMPureXP reagent (Beckman Coulter, Beverly, MA, USA). Quantity and quality of prepared libraries were assessed by Qubit^®^ 3.0 fluorometer (Thermofisher Scientific) and Agilent 2100 Bioanalyzer on-chip electrophoresis (Agilent Technologies), respectively, as previously described [41]. Subsequently, libraries were mixed in an equimolar ratio and emulsion PCR was performed with the Ion OneTouch OT2 System (Thermofisher Scientific) using Ion 540 Kit-OT2 (Thermofisher Scientific). Finally, sequencing was performed with Ion 540 Chip (Thermofisher Scientific) using Ion Torrent S5 (Thermofisher Scientific) instrument. The sequencing data was analyzed with Amplicon Suite (SmartSeq s.r.l.) and Ion Reporter Software v.5.12 (Thermofisher Scientific).

### 4.3. Sanger Sequencing

Genetic variants detected in the analyzed genes were validated by Sanger sequencing using a BigDye Therminator 3.1 Cycle Sequencing Kit (Life Technologies, Carlsbad, CA, USA) and read through the 3130xl Genetic Analyzer (Applied Biosystems, Foster City, CA, USA), according to manufacturer’s protocols.

### 4.4. Genetic Variant Classification

Based on the classification criteria developed by the Evidence-based Network for the Interpretation of Germline Mutant Alleles (ENIGMA) consortium (https://enigmaconsortium.org/) and according to IARC recommendations [26], the detected genetic variants were divided into five classes: benign (class I), likely benign (class II), variant of uncertain significance (VUS, class III), likely pathogenic (class IV), and pathogenic (class V). For the identification and classification of genetic variants, several databases, such as ClinVar, *BRCA* Exchange, LOVD, were used. The localization of the variants on the different genetically tested genes was described and graphically represented using the informatic tool Mutation Mapper-cBioPortal for Cancer Genomics [42,43].

The variants identified in the different genes were named according to the recommendations for the description of sequence variants provided by the Human Genome Variation Society (HGVS). HGVS nomenclature was approved by the HGVS, Human Variome Project (HVP), and the Human Genome Organization (HUGO) [44].

## 5. Conclusions

In this work, we focused on potential clinical impact of a NGS-based multi-gene panel testing in BBC individuals, in order to assess the utility of performing a most comprehensive genetic analysis in these patients, regardless the criteria concerning personal and family history of cancer established by the current guidelines.

Our study revealed that the use of a NGS-based multiple-gene panel testing could improve the detection rates of germline deleterious alterations in patients affected by BBC, since we observed that, in the absence of an analysis performed via multi-gene panel, a significant proportion (14.4%) of PVs would have been lost.

## Figures and Tables

**Table 1:** Clinical characteristics of bilateral breast cancer patients.

BBC (*n* = 139)	No. Patients (%)
**Tumor description**	
Metachronous	93 (66.9%)
Synchronous	46 (33.1%)
**Sex**	
**Female**	137
**Male**	2
**Age at diagnosis (years)**	
**Primary tumor**	
**Median (range)**	45 (21–77)
≤40	41 (29.5%)
41–50	53 (38.12%)
51–60	29 (20.9%)
≥60	16 (11.5%)
**Secondary tumor**	
**Median (range)**	51 (21–80)
≤40	21 (15.1%)
41–50	48 (34.5%)
51–60	40 (28.8%)
≥60	30 (21.6%)
**Time between 1st and 2nd tumors (years)**	
**Median ± DS**	4 ± 6.08
≤5	88 (63.3%)
6–10	27 (19.4%)
>10	24 (17.3%)
**Histology 1st breast cancer**	
Ductal in situ	96 (69%)
Ductal invasive	13 (9.4%)
Lobular invasive	12 (8.6%)
Others	18 (13%)
**Histology 2nd breast cancer**	
Ductal in situ	16 (11.5%)
Ductal invasive	92 (66.2%)
Lobular invasive	15 (10.8%)
Others	16 (11.5%)
**Molecular phenotype 1st breast cancer**	
Luminal A	28 (20.1%)
Luminal B/HER2 -	49 (35.3%)
Luminal B/HER2 +	7 (5%)
HER2 + (non-luminal)	6 (4.3%)
Triple negative	12 (8.6%)
Unknown	37 (26.7%)
**Molecular phenotype 2nd breast cancer**	
Luminal A	35 (25.2%)
Luminal B/HER2 -	35 (25.2%)
Luminal B/HER2 +	10 (7.2%)
HER2 + (non-luminal)	5 (3.6%)
Triple negative	17 (12.2%)
Unknown	37 (26.6%)
**Genetic testing results**	
Pathogenic variant *BRCA1*	13 (9.3%)
Pathogenic variant *BRCA2*	19 (13.7%)
Pathogenic variant *BRCA1/BRCA2*	1 (0.7%)
Pathogenic variant *CHEK2*	5 (3.6%)
Pathogenic variant *PALB2*	8 (5.8 %)
Pathogenic variant *ATM*	3 (2.2%)
Pathogenic variant *PTEN*	1 (0.7%)
Pathogenic variant *RAD51C*	2 (1.4%)
Pathogenic variant *MUTHY*	1 (0.7%)
Variant of uncertain significance	27 (19.4%)
Negative	59 (42.5%)
**Type of surgery 1st breast cancer**	
Mastectomy	50 (36.2%)
Breast conserving therapy	74 (53.2%)
Unknown	15 (10.9%)
**Type of surgery 2nd breast cancer**	
Mastectomy	48 (34.5%)
Breast conserving therapy	55 (39.9%)
Unknown	35 (25.3%)

**Table 2:** *BRCA1/2* pathogenic variants detected in patients with bilateral breast cancers.

Gene	Variant Type	HGVS Nomenclature	Protein Change	No. Patients	Allele Frequency (ExAC */GnomAD **)	Allele Frequency (Study Cohort)
***BRCA1***	fs	c.4964_4982del	p.Ser1655fs	4 (7.4%)	ExAC 0.000008	0.0.14
***BRCA1***	fs	c.3226_3227AG [1]	p.Gly1077fs	2 (3.8%)	GnomAD 0.000004	0.0072
***BRCA1***	NS	c.3904G>T	p.Glu1302Ter	2 (3.8%)	/	0.0072
***BRCA1***	fs	c.1531del	p.Gly512Terfs	1 (1.9%)	/	0.0036
***BRCA1***	fs	c.514del	p.Gln172fs	1 (1.9%)	ExAC 0.000008 GnomAD 0.000004	0.0036
***BRCA1***	fs	c.5266dupC	p.Gln1756Profs	1 (1.9%)	ExAC 0.000156 GnomAD 0.00018	0.0036
***BRCA1***	fs	c.3266del	p.Leu1089fs	1 (1.9%)	/	0.0036
***BRCA1***	NS	c.4327C > T	p.Arg1443Ter	1 (1.9%)	GnomAD 0.000024	0.0036
***BRCA2***	fs	c.1238del	p.Leu413fs	4 (7.4%)	GnomAD 0.000004	0.0.14
***BRCA2***	fs	c.9026_9030del	p.Tyr3009fs	2 (3.8%)	GnomAD 0.000004	0.0072
***BRCA2***	fs	c.9253dupA	p.Thr3085Asnfs	2 (3.8%)	/	0.0072
***BRCA2***	fs	c.6082_6086del	p.Glu2028fs	2 (3.8%)	ExAC 0.000008 GnomAD 0.000004	0.0072
***BRCA2***	fs	c.9098_9099insA	p.Gln3034fs	1 (1.9%)	/	0.0036
***BRCA2***	NS	c.8594T > A	p.Leu2865Ter	1 (1.9%)	/	0.0036
***BRCA2***	M	c.631G > A	p.Val211Ile	1 (1.9%)	/	0.0036
***BRCA2***	fs	c.5851_5854del	p.Ser1951fs	1 (1.9%)	/	0.0036
***BRCA2***	fs	c.2808_2811del	p.Ala938Profs	1 (1.9%)	ExAC 0.000017 GnomAD 0.000008	0.0036
***BRCA2***	IVS	c.7008-2A > T	/	1 (1.9%)	/	0.0036
***BRCA2***	IVS	c.476-2A > G	/	1 (1.9%)	/	0.0036
***BRCA2***	M	c.7007G > A	p.Arg2336His	1 (1.9%)	/	0.0036
***BRCA2***	NS	c.93G > A	p.Trp31Ter	1 (1.9%)	GnomAD 0.000004	0.0036
***BRCA2/*** ***BRCA1***	IVS/M	c.8331 + 2T > C/c.181T > G	/p.Cys61Gly	1 (1.9%)	/-ExAC 0.000067 GnomAD 0.000032	0.0036

Abbreviations: fs, frameshift; IVS, intronic variants; M, missense; NS, non-sense. * Dataset ExAC v1.0; ** Dataset GnomAD v2.1.1.

**Table 3:** Pathogenic variants detected in no-*BRCA* genes of patients with bilateral breast cancers positively tested by multi-gene panel.

Gene	Variant Type	HGVS Nomenclature	Protein Change	No. Patients	Allele Frequency (ExAC */GnomAD **)	Allele Frequency (Study Cohort)
***CHEK2***	fs	c.1100del	p.Thr367fs	5 (9.2%)	gnomAD 0.00204 ExAC 0.00182	0.018
***RAD51C***	NS	c.224dup	p.Tyr75Ter	1 (1.9%)	gnomAD 0.00001	0.0036
***ATM***	NS	c.8818_8821dup	p.Ser2941Ter	1 (1.9%)	/	0.0036
***PALB2***	fs	c.758dup	p.Ser254fs	2 (3.8%)	gnomAD 0.00002 ExAC 0.00003	0.0072
***PALB2***	NS	c.2566C > T	p.Gln856Ter	2 (3.8%)	gnomAD 0.00000 ExAC 0.00001	0.0072
***PALB2***	fs	c.1050_1053del	p.Thr351fs	2 (3.8%)	/	0.0072
***PALB2***	NS	c.2257C > T	p.Arg753Ter	2 (3.8%)	gnomAD 0.00002 ExAC 0.00003	0.0072
***MUTYH***	M	c.1103G > A	p.Gly368Asp	1 (1.9%)	gnomAD 0.00303 ExAC 0.00280	0.0036
***PTEN***	M	c.284C > A	p.Pro95Gln	1 (1.9%)	/	0.0036
***ATM***	M	c.8147T > C	p.Val2716Ala	1 (1.9%)	gnomAD 0.00003 ExAC 0.00004	0.0036
***RAD51C***	IVS	c.1026 + 5_1026 + 7del	/	1 (1.9%)	/	0.0036
***ATM***	LGR	Exon 57–61del	/	1 (1.9%)	/	0.0036

Abbreviations: fs, frameshift; IVS, intronic variants; LGR, large genomic rearrangement; M, missense; NS, non-sense. *Dataset ExAC v1.0; ** Dataset GnomAD v2.1.1.

**Table 4:** Variant of uncertain significance detected in patients with bilateral breast cancers.

Gene	Variant Type	HGVS Nomenclature	Protein Change	Variant Interpretation	No. Patients	Allele Frequency (ExAC ^a^/GnomAD ^b^)	Allele Frequency (Cohort Study)
***BRCA1***	M	c.4054G > A	p.Glu1352Lys	VUS	1 (3.70%)	gnomAD 0.00002 ExAC 0.00004	0.0036
***BRCA1***	IVS	c.670 + 31A > C	/	VUS	1 (3.70%)	/	0.0036
***BRCA2***	M	c.5267T > A	p.Val1756Glu	VUS	1 (3.70%)	gnomAD 0.00000 ExAC 0.00001	0.0036
***BRCA2***	M	c.8299C > T	p.Pro2767Ser	VUS	1 (3.70%)	/	0.0036
***BRCA2***	M	c.1769T > G	p.Phe590Cys	CIP	1 (3.70%)	gnomAD 0.00001 ExAC 0.00002	0.0036
***BRCA2***	M	c.9581C > A	p.Pro3194Gln	CIP	1 (3.70%)	gnomAD 0.00001 ExAC 0.00001	0.0036
***TP53***	M	c.446C > T *	p.Ser149Phe	VUS	1 (3.70%)	/	0.0036
***ATM***	M	c.1229T > C *	p.Val410Ala	CIP		gnomAD 0.00222 ExAC 0.00217	0.0036
***EPCAM***	M	c.334G > A	p.Gly112Ser	VUS	1 (3.70%)	/	0.0036
***MUTYH***	M	c.1378 C > T **	p.Arg460Cys	VUS	1 (3.70%)	gnomAD 0.00005 ExAC 0.00005	0.0036
***NBN***	M	c.283G > A **	p.Asp95Asn	CIP		gnomAD 0.00173 ExAC 0.00186	0.0036
***ATM***	M	c.6407G > C	p.Arg2136Thr	VUS	1 (3.70%)	/	0.0036
***MUTYH***	M	c.202T > C	p.Ser68Pro	VUS	1 (3.70%)	gnomAD 0.00001 ExAC 0.00001	0.0036
***CHEK2***	M	c.1388G > A	p.Cys463Tyr	VUS	2 (7.4%)	gnomAD 0.00000	0.0072
***MSH2***	M	c.1111G > C	p.Glu371Gln	VUS	1 (3.70%)	/	0.0036
***APC***	M	c.3920T > A	p.Ile1307Lys	CIP, Risk Factor	1 (3.70%)	gnomAD 0.00201 ExAC 0.00169	0.0036
***ATM***	M	c.4060C > A ***	p.Pro1354Thr	CIP	1 (3.70%)	gnomAD 0.00019 ExAC 0.00021	0.0036
***CHEK2***	M	c.1441 G > T ***	p.Asp481Tyr	CIP	2 (7.4%)	gnomAD 0.00039 ExAC 0.00028	0.0072
***ATM***	M	c.6983C > T	p.Pro2328Leu	VUS	1 (3.70%)	gnomAD 0.00000	0.0036
***APC***	M	c.3949G > C ****	p.Glu1317Gln	CIP	1 (3.70%)	gnomAD 0.00438 ExAC 0.00413	0.0036
***ATM***	M	c.4258C > T ****	p.Leu1420Phe	CIP		gnomAD 0.01104 ExAC 0.01271	0.0036
***BARD1***	M	c.1793C > T	p.Thr598Ile	VUS	1 (3.70%)	gnomAD 0.000008	0.0036
***ATM***	M	c.6067G > A	p.Gly2023Arg	CIP	1 (3.70%)	gnomAD 0.00143 ExAC 0.00157	0.0036
***MSH6***	M	c.663A > C	p.Glu221Asp	CIP	2 (7.4%)	gnomAD 0.00071 ExAC 0.00063	0.0072
***ATM***	M	c.6293T > C	p.Leu2098Pro	VUS	1 (3.70%)	gnomAD 0.00000 ExAC 0.00001	0.0036
***RAD50***	M	c.1277A > G	p.Gln426Arg	CIP	1 (3.70%)	gnomAD 0.00014 ExAC 0.00015	0.0036
***APC***	M	c.5338C > T	p.Pro1780Ser	VUS	1 (3.70%)	/	0.0036
***RAD50***	M	c.1094G > A	p.Arg365Gln	CIP	1 (3.70%)	gnomAD 0.00046 ExAC 0.00040	0.0036

Abbreviations: CIP, conflicting interpretations of pathogenicity; M, missense; VUS, variant of uncertain significance; ^a^ Dataset ExAC v1.0; ^b^ Dataset GnomAD v2.1.1 * These variants in *ATM* and *TP53* genes were observed in the same patient. ** These variants in *MUTYH* and *NBN* genes were observed in the same patient. *** These variants in *ATM* and *CHEK2* genes were observed in the same patient. **** These variants in *ATM* and *APC* genes were observed in the same patient.

## References

[1] Pan B., Xu Y., Zhou Y.D., Yao R., Wu H.W., Zhu Q.L., Wang C.J., Mao F., Lin Y., Shen S.J. (2019). The Prognostic Comparison Among Unilateral, Bilateral, Synchronous Bilateral, and Metachronous Bilateral Breast Cancer: A Meta-Analysis of Studies from Recent Decade (2008–2018). Cancer Med..

[2] Sim Y., Tan V.K.M., Sidek N.A.B., Chia D.K.A., Tan B.K.T., Madhukumar P., Yong W.S., Wong C.Y., Ong K.W. (2018). Bilateral Breast Cancers in An Asian Population, and A Comparison Between Synchronous and Metachronous Tumours. ANZ J. Surg..

[3] Lu W., Schaapveld M., Jansen L., Bagherzadegan E., Sahinovic M.M., Baas P.C., Hanssen L.M.H.C., van Der Mijle H.C.J., Brandenburg J.D., Wiggers T. (2009). The Value of Surveillance Mammography of the Contralateral Breast in Patients with a History of Breast Cancer. Eur. J. Cancer.

[4] Hartman M., Czene K., Reilly M., Adolfsson J., Bergh J., Adami H.-O., Dickman P.W., Hall P. (2007). Incidence and Prognosis of Synchronous and Metachronous Bilateral Breast Cancer. J. Clin. Oncol..

[5] Corredor J., Woodson A.H., Gutierrez Barrera A., Arun B. (2020). Multigene Panel Testing Results in Patients with Multiple Breast Cancer Primaries. Breast J..

[6] Apostolou P., Fostira F. (2013). Hereditary Breast Cancer: The Era of New Susceptibility Genes. Biomed Res. Int..

[7] Valencia O.M., Samuel S.E., Viscusi R.K., Riall T.S., Neumayer L.A., Aziz H. (2017). The Role of Genetic Testing in Patients with Breast Cancer. JAMA Surg..

[8] Kotsopoulos J., Huzarski T., Gronwald J., Singer C.F., Moller P., Lynch H.T., Armel S., Karlan B., Foulkes W.D., Neuhausen S.L. (2017). Bilateral Oophorectomy and Breast Cancer Risk Inbrca1andbrca2mutation Carriers. JNCI J. Natl. Cancer Inst..

[9] Miki Y., Swensen J., Shattuck-Eidens D., Futreal P., Harshman K., Tavtigian S., Liu Q., Cochran C., Bennett L., Ding W. (1994). A Strong Candidate for the Breast and Ovarian Cancer Susceptibility Gene Brca1. Science.

[10] Wooster R., Bignell G., Lancaster J., Swift S., Seal S., Mangion J., Collins N., Gregory S., Gumbs C., Micklem G. (1995). Identification of the Breast Cancer Susceptibility Gene Brca2. Nature.

[11] Kuchenbaecker K.B., Hopper J.L., Barnes D.R., Phillips K.-A., Mooij T.M., Roos-Blom M.-J., Jervis S., van Leeuwen F.E., Milne R.L., Andrieu N. (2017). Risks of Breast, Ovarian, and Contralateral Breast Cancer for Brca1 and Brca2 Mutation Carriers. JAMA.

[12] Metcalfe K., Gershman S., Lynch H.T., Ghadirian P., Tung N., Kim-Sing C., Olopade O.I., Domchek S., Mclennan J., Eisen A. (2011). Predictors of Contralateral Breast Cancer in Brca1 and Brca2 Mutation Carriers. Br. J. Cancer.

[13] Graeser M.K., Engel C., Rhiem K., Gadzicki D., Bick U., Kast K., Froster U.G., Schlehe B., Bechtold A., Arnold N. (2009). Contralateral Breast Cancer Risk in Brca1 and Brca2 Mutation Carriers. J. Clin. Oncol..

[14] Incorvaia L., Fanale D., Badalamenti G., Bono M., Calò V., Cancelliere D., Castiglia M., Fiorino A., Pivetti A., Barraco N. (2020). Hereditary Breast and Ovarian Cancer in Families from Southern Italy (Sicily)—Prevalence and Geographic Distribution of Pathogenic Variants in Brca1/2 Genes. Cancers.

[15] Thompson D. (2002). Cancer Incidence in Brca1 Mutation Carriers. J. Natl. Cancer Inst..

[16] Breast Cancer Linkage Consortium (1999). Cancer Risks in Brca2 Mutation Carriers. J. Natl. Cancer Inst..

[17] Bevers T.B., Helvie M., Bonaccio E., Calhoun K.E., Daly M.B., Farrar W.B., Garber J.E., Gray R., Greenberg C.C., Greenup R. (2018). Breast Cancer Screening and Diagnosis, Version 3.2018, Nccn Clinical Practice Guidelines in Oncology. J. Natl. Compr. Cancer Netw..

[18] Senkus E., Kyriakides S., Ohno S., Penault-Llorca F., Poortmans P., Rutgers E., Zackrisson S., Cardoso F. (2015). Primary Breast Cancer: Esmo Clinical Practice Guidelines for Diagnosis, Treatment and Follow-Up. Ann. Oncol..

[19] Tedaldi G., Tebaldi M., Zampiga V., Danesi R., Arcangeli V., Ravegnani M., Cangini I., Pirini F., Petracci E., Rocca A. (2017). Multiple-Gene Panel Analysis in A Case Series of 255 Women with Hereditary Breast and Ovarian Cancer. Oncotarget.

[20] Slavin T.P., Maxwell K.N., Lilyquist J., Vijai J., Neuhausen S.L., Hart S.N., Ravichandran V., Thomas T., Maria A., Villano D. (2017). The Contribution of Pathogenic Variants in Breast Cancer Susceptibility Genes to Familial Breast Cancer Risk. NPJ Breast Cancer.

[21] Economopoulou P., Dimitriadis G., Psyrri A. (2015). Beyond Brca: New Hereditary Breast Cancer Susceptibility Genes. Cancer Treat. Rev..

[22] Singer C.F., Balmaña J., Bürki N., Delaloge S., Filieri M.E., Gerdes A.-M., Grindedal E.M., Han S., Johansson O., Kaufman B. (2019). Genetic Counselling and Testing of Susceptibility Genes for Therapeutic Decision-Making in Breast Cancer—An European Consensus Statement and Expert Recommendations. Eur. J. Cancer.

[23] Narod S.A. (2014). Bilateral Breast Cancers. Nat. Rev. Clin. Oncol..

[24] Zhang S., Phelan C.M., Zhang P., Rousseau F., Ghadirian P., Robidoux A., Foulkes W., Hamel N., Mccready D., Trudeau M. (2008). Frequency of the Chek2 1100delc Mutation Among Women with Breast Cancer: An international Study. Cancer Res..

[25] Broeks A., De Witte L., Nooijen A., Huseinovic A., Klijn J.G.M., van Leeuwen F.E., Russell N.S., van’t Veer L.J. (2004). Excess Risk for Contralateral Breast Cancer in Chek2*1100delc Germline Mutation Carriers. Breast Cancer Res. Treat..

[26] Plon S.E., Eccles D.M., Easton D., Foulkes W.D., Genuardi M., Greenblatt M.S., Hogervorst F.B.L., Hoogerbrugge N., Spurdle A.B., Tavtigian S.V. (2008). Sequence Variant Classification and Reporting: Recommendations for Improving the Interpretation of Cancer Susceptibility Genetic Test Results. Hum. Mutat..

[27] Russo A., Calò V., Bruno L., Schirò V., Agnese V., Cascio S., Foddai E., Fanale D., Rizzo S., Di Gaudio F. (2008). Is Brca1-5083del19, Identified in Breast Cancer Patients of Sicilian Origin, a Calabrian Founder Mutation?. Breast Cancer Res. Treat..

[28] Janavičius R. (2010). Founder Brca1/2 Mutations in the Europe: Implications for Hereditary Breast-Ovarian Cancer Prevention and Control. EPMA J..

[29] Ding D., Zhang Y., He X., Meng W., Ma W., Zheng W. (2012). Frequency of the Chek2 1100delc Mutation among Women with Early-Onset and Bilateral Breast Cancer. Breast Cancer Res..

[30] Weischer M., Nordestgaard B.G., Pharoah P., Bolla M.K., Nevanlinna H., van’t Veer L.J., Garcia-Closas M., Hopper J.L., Hall P., Andrulis I.L. (2012). Chek2*1100delc Heterozygosity in Women with Breast Cancer Associated with Early Death, Breast Cancer–Specific Death, and Increased Risk of a Second Breast Cancer. J. Clin. Oncol..

[31] Stjepanovic N., Moreira L., Carneiro F., Balaguer F., Cervantes A., Balmaña J., Martinelli E. (2019). Hereditary Gastrointestinal Cancers: Esmo Clinical Practice Guidelines for Diagnosis, Treatment and Follow-Up. Ann. Oncol..

[32] Padmanabhan N. (2015). Synchronous Bilateral Breast Cancers. J. Clin. Diagn. Res..

[33] Huber A., Seidler S.J., Huber D.E. (2020). Clinicopathological Characteristics, Treatment and Outcome of 123 Patients with Synchronous or Metachronous Bilateral Breast Cancer in a Swiss Institutional Retrospective Series. Eur. J. Breast Health.

[34] Desmond A., Kurian A.W., Gabree M., Mills M.A., Anderson M.J., Kobayashi Y., Horick N., Yang S., Shannon K.M., Tung N. (2015). Clinical Actionability of Multigene Panel Testing for Hereditary Breast and Ovarian Cancer Risk Assessment. JAMA Oncol..

[35] Tung N., Battelli C., Allen B., Kaldate R., Bhatnagar S., Bowles K., Timms K., Garber J.E., Herold C., Ellisen L. (2015). Frequency of Mutations in Individuals with Breast Cancer Referred Forbrca1 and brca2 testing Using Next-Generation Sequencing with a 25-Gene Panel. Cancer.

[36] Easton D.F., Pharoah P.D.P., Antoniou A.C., Tischkowitz M., Tavtigian S.V., Nathanson K.L., Devilee P., Meindl A., Couch F.J., Southey M. (2015). Gene-Panel Sequencing and the Prediction of Breast-Cancer Risk. N. Engl. J. Med..

[37] Shin H.-C., Lee H.-B., Yoo T.-K., Lee E.-S., Kim R.N., Park B., Yoon K.-A., Park C., Lee E.S., Moon H.-G. (2020). Detection of Germline Mutations in Breast Cancer Patients with Clinical Features of Hereditary Cancer Syndrome Using A Multi-Gene Panel Test. Cancer Res. Treat..

[38] Crawford B., Adams S.B., Sittler T., van den Akker J., Chan S., Leitner O., Ryan L., Gil E., van ’t Veer L. (2017). Multi-Gene Panel Testing for Hereditary Cancer Predisposition in Unsolved High-Risk Breast and Ovarian Cancer Patients. Breast Cancer Res. Treat..

[39] Kurian A.W., Hare E.E., Mills M.A., Kingham K.E., Mcpherson L., Whittemore A.S., Mcguire V., Ladabaum U., Kobayashi Y., Lincoln S.E. (2014). Clinical Evaluation of a Multiple-Gene Sequencing Panel for Hereditary Cancer Risk Assessment. J. Clin. Oncol..

[40] Laduca H., Stuenkel A.J., Dolinsky J.S., Keiles S., Tandy S., Pesaran T., Chen E., Gau C.-L., Palmaer E., Shoaepour K. (2014). Utilization of Multigene Panels in Hereditary Cancer Predisposition Testing: Analysis of More Than 2000 Patients. Genet. Med..

[41] Hoheisel J.D., Simbolo M., Gottardi M., Corbo V., Fassan M., Mafficini A., Malpeli G., Lawlor R.T., Scarpa A. (2013). DNA Qualification Workflow for Next Generation Sequencing of Histopathological Samples. PLoS ONE.

[42] Gao J., Aksoy B.A., Dogrusoz U., Dresdner G., Gross B., Sumer S.O., Sun Y., Jacobsen A., Sinha R., Larsson E. (2013). Integrative Analysis of Complex Cancer Genomics and Clinical Profiles Using the Cbioportal. Sci. Signal..

[43] Cerami E., Gao J., Dogrusoz U., Gross B.E., Sumer S.O., Aksoy B.A., Jacobsen A., Byrne C.J., Heuer M.L., Larsson E. (2012). The Cbio Cancer Genomics Portal: An Open Platform for Exploring Multidimensional Cancer Genomics Data: Figure 1. Cancer Discov..

[44] Den Dunnen J.T., Dalgleish R., Maglott D.R., Hart R.K., Greenblatt M.S., Mcgowan-Jordan J., Roux A.-F., Smith T., Antonarakis S.E., Taschner P.E.M. (2016). Hgvs Recommendations for the Description of Sequence Variants: 2016 Update. Hum. Mutat..

